# Spontaneous Polymicrobial Empyema in an Immunocompetent Patient: The Role of Imaging Accuracy

**DOI:** 10.7759/cureus.79482

**Published:** 2025-02-22

**Authors:** Ipsit Shah, Mukul Sharda, Pinky Jha, Abhijai Singh

**Affiliations:** 1 Internal Medicine, Medical College of Wisconsin, Milwaukee, USA

**Keywords:** empyema necessitasis, parvimonas, pleural effusion, prevotella, streptococcus anginosus group

## Abstract

Empyema, a bacterial infection in the pleural space, is a subtype of complicated parapneumonic effusion. It is best identified through clinical presentation, imaging (chest X-ray, ultrasound, and CT), and microbiological confirmation. We present the case of a 20-year-old female with a history of bronchiectasis who developed spontaneous polymicrobial empyema. Upon arrival, the patient had an oxygen saturation of 93% on room air but later developed tachypnea and tachycardia. Imaging revealed a trapped lung with associated empyema and gas bubbles rather than a true hydropneumothorax. Cultures tested positive for *Streptococcus anginosus*,* Prevotella intermedia*, and *Parvimonas micra*. The patient underwent pulmonary decortication surgery and therapeutic bronchoscopy, received a four-week course of IV piperacillin-tazobactam, and showed significant improvement upon follow-up. This case underscores the importance of accurate radiologic interpretation and early multidisciplinary intervention in high-risk patients. It highlights the diagnostic challenge of differentiating a trapped lung with empyema from hydropneumothorax, emphasizing the role of precise imaging analysis in clinical decision-making.

## Introduction

Empyema, a subtype of pleural effusion, is a condition caused by bacterial infections in the chest cavity. It is identified by the presence of pus in the pleural space and falls under the category of complicated parapneumonic effusion. The development of empyema in patients with community-acquired pneumonia (CAP) is associated with several risk factors, including a history of alcohol abuse, intravenous drug use, and conditions predisposing to aspiration, such as neurological diseases. Conversely, certain studies have identified factors like chronic obstructive pulmonary disease (COPD) as having a varied impact on the likelihood of developing empyema [1].

Empyema continues to grow in frequency globally and remains a significant source of health problems and death in both developed and developing countries. The causes of empyema may differ based on region and patient characteristics and have altered over time. In Europe and North America, the *Streptococcus anginosus* group is the most frequently identified cause of empyema, while *Klebsiella pneumoniae* is predominant in Taiwan. A retrospective analysis of a database of patients with empyema admitted to Kurashiki Central Hospital in Japan between 2007 and 2015 revealed that the combinations of microorganisms present in polymicrobial empyema were predominantly members of the *S. anginosus* group and anaerobes, with the second most common being a mixture of other aerobic and anaerobic isolates [2]. Patients with polymicrobial empyema are more likely to experience a poor outcome compared to those with monomicrobial empyema [2].

We report the case of a 20-year-old female who presented to the emergency department (ED) with right-sided chest pain with worsening shortness of breath, subsequently diagnosed with the presence of spontaneous polymicrobial empyema.

## Case presentation

A 20-year-old female patient with a history of bronchiectasis presented with right-sided chest pain and worsening shortness of breath for two days. She had previously been treated for pseudomonas pneumonia with cefepime and levofloxacin five months prior, which resolved. Oxygen saturation was 93% on room air at presentation, and she later developed tachypnea and tachycardia.

**Figure 1:**
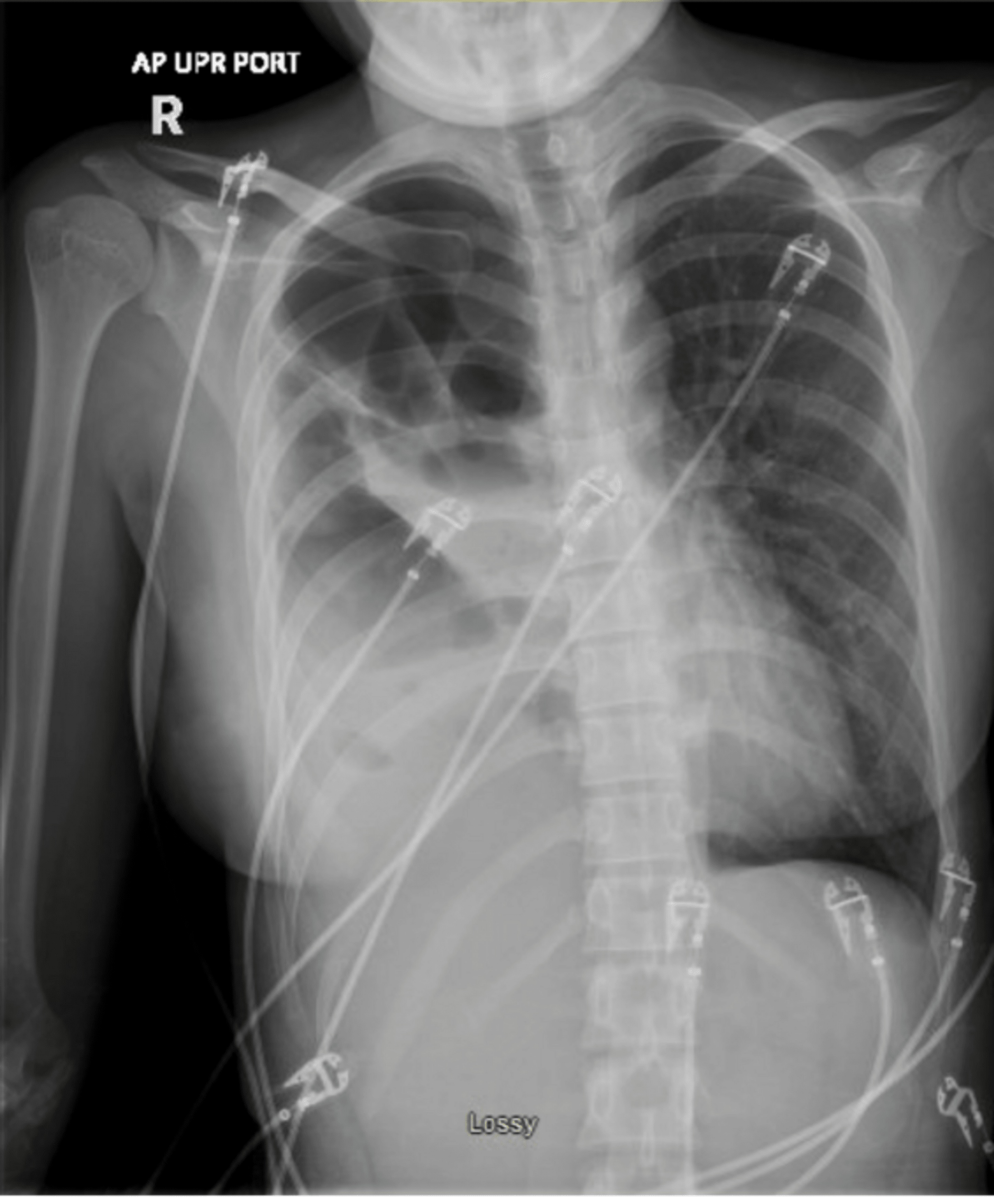
Chest X-ray showing a right-sided pleural effusion with an associated trapped lung.

**Figure 2:**
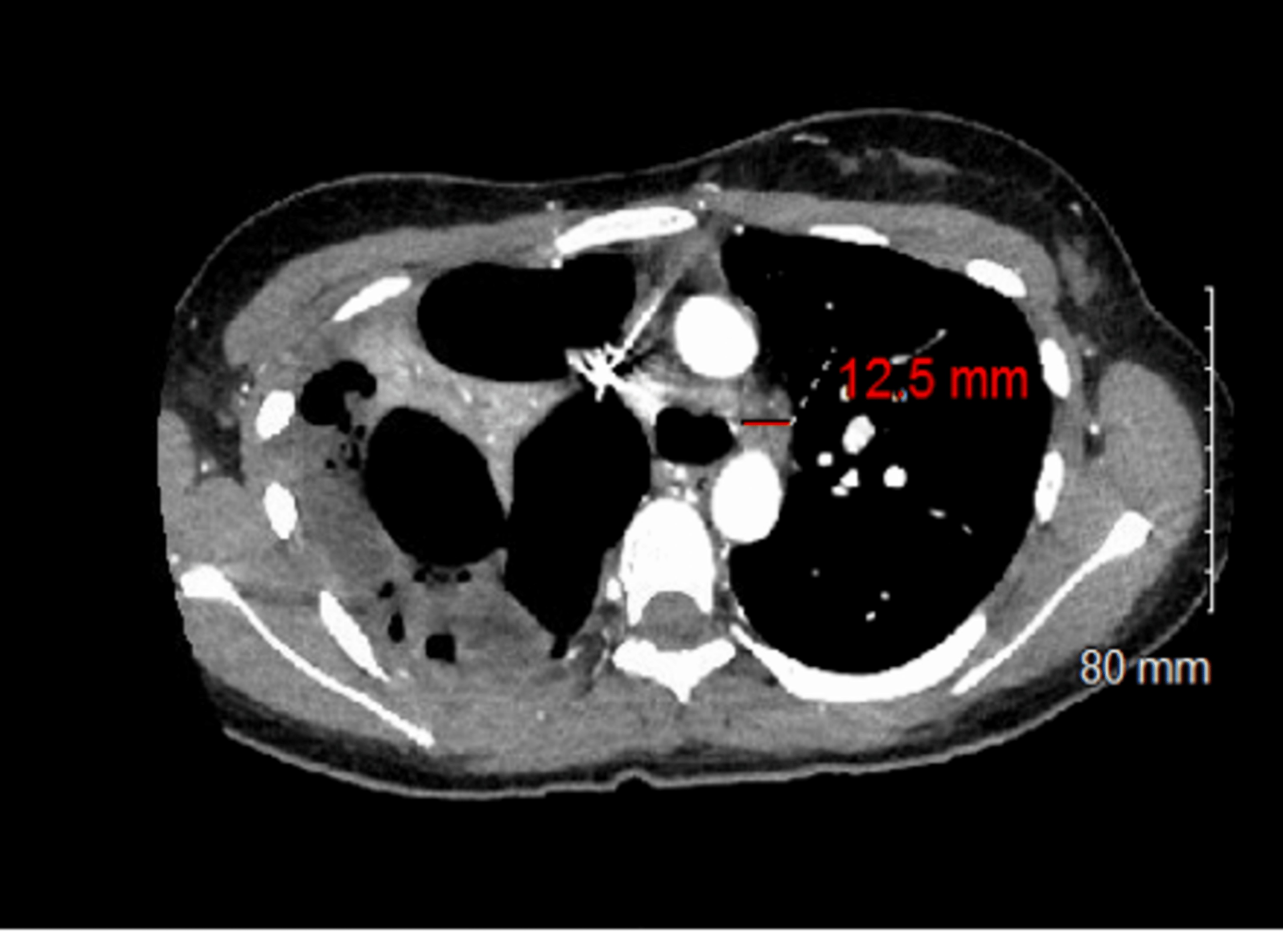
CT scan demonstrating occlusion of the right mainstem bronchus, right lung collapse, and multiloculated pleural collection with gas bubbles.

A chest X-ray showed a right-sided pleural effusion with right lung volume loss (Figure 1). CT imaging revealed occlusion of the right mainstem bronchus with a new right lung collapse, architectural distortion, and a multiloculated collection with gas bubbles, indicative of empyema with a trapped lung (Figure 2). A pigtail catheter initially drained 1200 mL of purulent fluid. Laboratory results were unremarkable except for an elevated ESR of 44 mm/hour (normal: 0-28 mm/hour). Cultures from the pleural fluid showed 1+ *Streptococcus anginosus*, 4+ *Prevotella intermedia*, and 4+ *Parvimonas micra *(Table 1).

**Table 1:** Microbiological analysis results of pleural fluid. The microbiological analysis of pleural fluid revealed a polymicrobial infection with *Streptococcus anginosus*, *Prevotella intermedia*, and *Parvimonas micra*, supporting the diagnosis of polymicrobial empyema. PMNs: polymorphonuclear leukocytes; Aer/Ana: aerobic and anaerobic

Aer/Ana Culture	Gram Stain
1+ *Streptococcus anginosus*	3+ PMNs
4+ *Prevotella intermedia*	4+ Gram-negative rods
4+ *Parvimonas* (*Peptostreptococcus*) *micra*	4+ Gram-positive cocci

The patient underwent pulmonary decortication surgery and therapeutic bronchoscopy to remove the pleural rind and mobilize the right lung. Two large-bore chest tubes were placed postoperatively, draining 480 mL of reddish-clear fluid. No air leak was observed. Infectious disease specialists recommended a four-week course of IV piperacillin-tazobactam. A peripherally inserted central catheter (PICC) line was placed, and the patient was discharged with scheduled follow-up and repeat imaging.

At the one-month follow-up, the patient demonstrated significant clinical improvement, with resolution of pain and improved right upper lung visualization on chest X-ray. The PICC line was subsequently removed.

## Discussion

Polymicrobial empyema is more commonly observed in immunosuppressed individuals and is associated with higher morbidity. Our case emphasizes the importance of considering polymicrobial infections even in young immunocompetent individuals, particularly those with underlying structural lung disease.

Radiographic interpretation is crucial in differentiating a trapped lung with empyema from hydropneumothorax. In this case, the presence of gas bubbles within the empyema was due to gas-producing organisms rather than pneumothorax. Misinterpretation of imaging findings can impact clinical decision-making and influence management strategies.

Management of polymicrobial empyema includes prompt antibiotic therapy, drainage, and, in selected cases, surgical intervention. Intrapleural fibrinolytic therapy has been found beneficial in reducing surgical interventions but does not significantly impact mortality. Our patient's successful recovery was attributed to a multidisciplinary approach, including respiratory, infectious disease, and surgical teams. While this case follows standard treatment guidelines, it serves as an educational case emphasizing the importance of early imaging review and accurate interpretation.

Previous studies have highlighted similar cases of polymicrobial empyema involving anaerobic bacteria, including *Parvimonas micra* and *Prevotella intermedia* [3,4]. These organisms have been implicated in pleural infections and are commonly associated with orodental flora, which may play a role in spontaneous empyema in select patients. Additionally, the role of intrapleural fibrinolytic therapy has been explored in managing empyema [5], demonstrating its effectiveness in select cases where surgical intervention is not immediately indicated.

## Conclusions

Physicians should maintain a high index of suspicion for polymicrobial empyema, particularly in patients with structural lung disease. Accurate radiological interpretation, prompt microbiological assessment, and timely surgical intervention can significantly improve patient outcomes. While this case does not introduce a novel treatment, it underscores the importance of recognizing radiologic pitfalls and ensuring timely multidisciplinary management. The diagnostic challenge in distinguishing trapped lung with empyema from hydropneumothorax highlights the need for precise imaging interpretation to avoid misclassification and guide appropriate therapeutic strategies.
